# Novel Respiratory Syncytial Virus-Like Particle Vaccine Composed of the Postfusion and Prefusion Conformations of the F Glycoprotein

**DOI:** 10.1128/CVI.00720-15

**Published:** 2016-06-06

**Authors:** Velasco Cimica, Hélène Boigard, Bipin Bhatia, John T. Fallon, Alexandra Alimova, Paul Gottlieb, Jose M. Galarza

**Affiliations:** a TechnoVax, Inc., Tarrytown, New York, USA; b EMD-Millipore Corporation, Billerica, Massachusetts, USA; c Department of Pathology, New York Medical College, Westchester Medical Center, Valhalla, New York, USA; d Department of Pathobiology, Sophie Davis School of Biomedical Education, City College of New York, New York, New York, USA; University of Maryland School of Medicine

## Abstract

Respiratory syncytial virus (RSV) is the leading cause of severe respiratory disease in infants and children and represents an important global health burden for the elderly and the immunocompromised. Despite decades of research efforts, no licensed vaccine for RSV is available. We have developed virus-like particle (VLP)-based RSV vaccines assembled with the human metapneumovirus (hMPV) matrix protein (M) as the structural scaffold and the RSV fusion glycoprotein (F) in either the postfusion or prefusion conformation as its prime surface immunogen. Vaccines were composed of postfusion F, prefusion F, or a combination of the two conformations and formulated with a squalene-based oil emulsion as adjuvant. Immunization with these VLP vaccines afforded full protection against RSV infection and prevented detectable viral replication in the mouse lung after challenge. Analyses of lung cytokines and chemokines showed that VLP vaccination mostly induced the production of gamma interferon (IFN-γ), a marker of the Th1-mediated immune response, which is predominantly required for viral protection. Conversely, immunization with a formalin-inactivated RSV (FI-RSV) vaccine induced high levels of inflammatory chemokines and cytokines of the Th2- and Th17-mediated types of immune responses, as well as severe lung inflammation and histopathology. The VLP vaccines showed restricted production of these immune mediators and did not induce severe bronchiolitis or perivascular infiltration as seen with the FI-RSV vaccine. Remarkably, analysis of the serum from immunized mice showed that the VLP vaccine formulated using a combination of postfusion and prefusion F elicited the highest level of neutralizing antibody and enhanced the Th1-mediated immune response.

## INTRODUCTION

**FIG 1:**
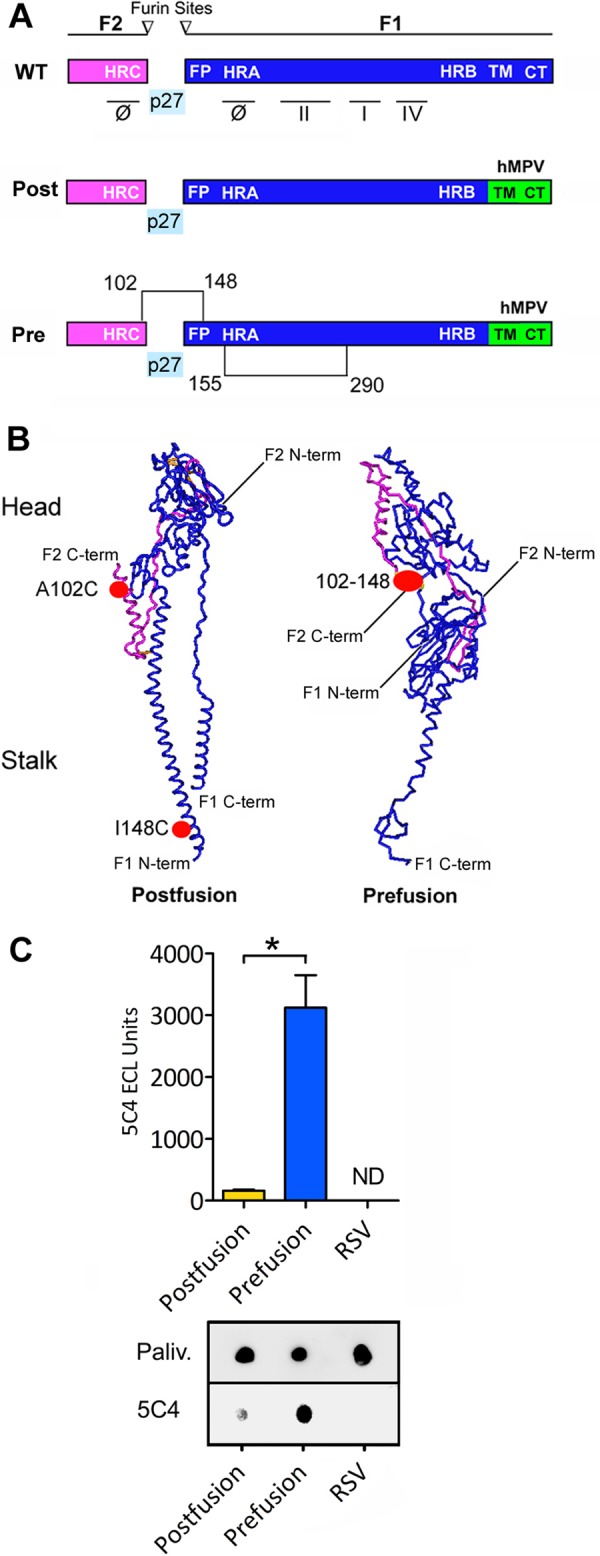
Development of RSV F constructs using structural vaccinology. (A) Schematic representation of the wild-type (WT) RSV F primary structure. F protein matures by furin enzyme cleavage at sites I and II, generating the F2-F1 protomer and releasing p27 glycopeptide. F protein is characterized by the heptad repeat domains HRA, HRB, and HRC, fusion peptide (FP), transmembrane domain (TM), and cytosolic tail (CT), which is important for virion assembly with the matrix M protein. F elicits neutralizing antibodies able to recognize the antigenic sites: Φ, I, II, and IV. The figure includes a schematic picture of the postfusion hybrid construct (Post) with the CT swapped with the analogous domain of the hMPV F (green) and a schematic representation of prefusion hybrid construct (Pre) with modification of disulfide bonds 102 to 148 and 155 to 290. (B) Tridimensional structure representation of the F protomer in postfusion and prefusion conformations. The cysteine modifications A102C and I148C are indicated in red. The prefusion conformation is maintained by formation of a cysteine link between the F2 (purple) and F1 (blue) chains (the positions of amino acids 102 and 148 are approximated in the postfusion structure representation). (C) Dot blot analysis showing the immune reactivity of recombinant RSV F proteins. The graph represents the results from 3 independent experiments for 5C4 normalized over palivizumab immune reactivity, and a single dot blot experiment is shown. The asterisk indicates a statistically significant *P* value (*P* < 0.05) between the postusion and prefusion conditions. ND, not detected.

Human respiratory syncytial virus (RSV) is the leading cause of severe pediatric pulmonary disease worldwide. RSV infects nearly all infants at least once by the age of 2 years. Epidemiological studies around the globe indicate that 2 to 5% of the children infected with RSV require hospitalization, with the most severe morbidity and mortality disproportionally seen in premature infants. RSV disease causes 100,000 to 200,000 fatalities per year globally (1, 2). It is believed that severe RSV infection can predispose children to develop wheezing with future illnesses and potentially to develop asthma (3, 4). RSV infection elicits neutralizing antibodies and a T-cell response that wane over time; consequently, the patient is often unprotected against reinfection (5, 6). Furthermore, elderly people show a greater risk of severe RSV disease upon reinfection (7). Despite decades of research efforts, no licensed vaccine is currently available to control or prevent RSV infection (8). Vaccinology research shows that the F glycoprotein is the most attractive target for eliciting neutralizing antibodies against the virus. RSV displays different conformations of F that are antigenically distinct: the highly stable postfusion and the metastable prefusion (9). Magro et al. (10) have demonstrated that antibodies specific to prefusion F account for most of the neutralizing activity in a prophylactic human Ig preparation and immunized rabbits. Subsequently, McLellan and coworkers (9) determined the protein structure of the prefusion F by X-ray crystallography and identified the prefusion-only antigenic site Φ (Fig. 1A). While palivizumab can recognize both postfusion and prefusion structures, a subset of highly neutralizing antibodies (5C4, AM22, and D25) bind specifically to the prefusion antigenic site Φ (9, 10). Interestingly, the AM14 and MPE8 neutralizing antibodies are also able to very efficiently recognize the prefusion F using alternative antigenic sites. This demonstrates that the prefusion F expresses multiple epitopes suitable for target therapy (11, 12), which are not exhibited in the postfusion conformation.

Adopting structural vaccinology, our group has developed virus-like particle (VLP) vaccines containing recombinant postfusion and prefusion F hybrids together with the human metapneumovirus (hMPV) matrix protein (M). Efficacy studies showed that immunization with prefusion F VLP vaccine, postfusion F VLP vaccine, or a combination of the two VLP vaccines (combo VLP vaccine) afforded complete protection against an RSV challenge. Importantly, VLP vaccination was safe and effective in stimulating a Th1-type cytokine profile and the combo VLP vaccine elicited the highest level of IgG2a antibody and neutralization activity.

## MATERIALS AND METHODS

### Structural vaccinology.

The RSV fusion (F) (GenBank accession number ACO83302.1) and human metapneumovirus matrix (M) (GenBank accession no. AIY25728.1) genes were codon optimized and chemically synthesized (Blue Heron Biotech, WA). Prefusion F mutants were designed by protein structure analysis using Cn3D software (NCBI, MD) and data from the NCBI repository (9, 13). Wild-type optimized RSV F was subcloned into an expression vector and mutagenized by cysteine substitutions using the QuikChange II kit (Agilent, CA) and DNA oligonucleotides (IDT, TX). The cytoplasmic tail (CT) and the transmembrane domain (TM) of RSV F were swapped with hMPV F analog domains using recombinant DNA methods: the hRSV F sequence from amino acids 1 to 524 (GenBank accession no. ACO83302.1) was joined to the hMPV F sequence from amino acids 489 to 539 (GenBank accession no. AEK26895.1). Constructs were fully sequenced for quality verification.

### Vaccine.

RSV virus-like particles (VLPs) were produced in a suspension culture of mammalian cells following transient transfection of the VLP protein expression plasmids. Expi293F cells were maintained in serum-free Expi293 expression medium (Life Technologies, CA). The transfection reaction mixture was assembled in separate tubes: 1.0 μg of DNA vectors, 3.0 μg of 25-kDa linear polyethylenimine (PEI) (Polysciences, PA), and 0.1 ml of Opti-MEM, for each 1 ml of cell culture (2.5 × 10^6^ cells/ml). The plasmids for RSV F and hMPV M were transfected at ratios of 70% and 30%, respectively. The mixture was incubated for 15 min at room temperature and then added to the cells. Forty-eight hours after transfection, the culture supernatant was clarified by centrifugation at 8,000 × *g* for 10 min, and VLPs were concentrated by ultracentrifugation for 2 h at 140,000 × *g* using an SW 28 rotor (Beckman Coulter, CA). The pellets were resuspended in 250 μl of buffer E (250 mM sucrose, 100 mM MgSO_4_, 10 mM Tris [pH 7.5]) containing protease inhibitors (Thermo Scientific, MA). The VLPs were subsequently purified by ultracentrifugation through a continuous sucrose gradient (10% to 50%) for 4 h at 180,000 × *g* using an SW 40Ti rotor. The VLPs were collected from the 15% to 25% fraction and further purified by ultrafiltration using a Vivaspin system with a 100-kDa cutoff (Sartorious, NY). The VLPs were characterized by Western blotting, dot blotting, and electron microscopy. The combo VLP vaccine was prepared by blending equal amounts (2 μg F content) of VLPs with prefusion F and postfusion F per dose (4 μg total F content). The formalin-inactivated RSV (FI-RSV) vaccine was produced using the protocol described by Prince et al (14). The final preparation was adjuvanted with Imject alum (Thermo Scientific, MA) to a final concentration of 4 mg/ml of aluminum hydroxide and then diluted 1:25 in phosphate-buffered saline (PBS) for animal administration.

### Dot blotting and Western blotting.

The immune reactivity and antigen concentration of the VLP vaccine were assayed using a dot blot. The VLP samples in a volume of 1 to 5 μl were absorbed into nitrocellulose for 15 min and then blocked for 1 h with 3% nonfat dry milk in Tris-buffered saline plus 0.1% Tween 20 (TBS-Tween). The primary antibodies were palivizumab (Synagis; MedImmune, MD), 131-2a (EMD-Millipore, MA), 5C4 (9), and anti-hMVP M (clone 4821; GeneTex, CA). Horseradish peroxidase (HRP)-conjugated mouse anti-human antibody or goat anti-mouse antibody was used as the secondary antibody. Detection was performed using enhanced chemiluminescence (ECL) substrate and a digital imaging system (FluorChem M system; ProteinSimple, CA). The dot blot with palivizumab was used for quantifying the F content in the vaccines compared to a serial dilution of recombinant RSV F protein standard (Sino Biological, PA) using AlphaView SA imaging software (ProteinSimple). Western blotting was performed using a Novex gel system (Life Technologies) and NuPage 4 to 12% bis-Tris gel (Life Technologies).

### Electron microscopy analysis of the VLPs.

The VLPs were examined by negative staining and electron microscopy (Zeiss 902 transmission electron microscope with accelerating voltage of 80 kV). Five microliters of resuspended particles was applied to a Formvar-coated copper grid (FCF300-Cu; Electron Microscopy Sciences, PA) and stained with 2.0% phosphotungstic acid (pH 7.0). Immunogold labeling was performed as follows. First, 5 μl of resuspended samples was applied to a Formvar-coated grid and incubated for 5 min at room temperature (RT). Then the grid was washed 5 times with buffer (0.1% fetal bovine serum [FBS] in PBS, 10 mM glycine, 0.01% NaN_3_), fixed with 4% paraformaldehyde in PBS for 15 min, and blocked in 1% bovine serum albumin (BSA) in PBS for 30 min. The sample was floated onto 40 μl of palivizumab at a 1:1,000 dilution in 0.1% BSA in PBS plus 0.01% NaN_3_ buffer overnight at 4°C. The grid was then placed in a humid chamber to prevent evaporation. Following 5 washes with buffer, the grid was placed on a drop of 6-nm gold beads conjugated with goat anti-human polyclonal IgG antibody (Abcam, MA) at a 1:3 dilution in 0.1% BSA in PBS plus 0.01% NaN_3_ for 2 h at RT. Then the sample was washed 5 times in washing buffer and stained with 2.0% phosphotungstic acid (pH 7.0).

### Virus production and titration.

This study was performed in compliance with biosafety level 2 (BSL2) regulations. RSV strain A2 (ATCC VR-1540; ATCC, VA) was used for the murine challenge, production of the FI-RSV vaccine, immunization, and viral assays. RSV was propagated in HEp-2 cells (ATCC CCL-23; ATCC) using Dulbecco's modified Eagle's medium (DMEM) with 2% FBS (Life Technologies) as the culture medium, according to the supplier's protocol. Viral titration was performed by plaque assay using an HEp-2 monolayer. HEp-2 cells were infected for 1 hour for viral absorption with 10-fold serially diluted virus in serum-free DMEM, from a dilution range of 10^4^ to 10^8^. Subsequently, an overlay of 1% methylcellulose (C-4888; Sigma-Aldrich, MO) in DMEM supplemented with 2% FBS was applied to each well to prevent viral particle diffusion. After 4 to 5 days of incubation, the overlay was removed, and the cells were fixed using cold methanol for 20 min at −20°C. Virus plaques were stained by immunocytochemical techniques using an anti-RSV goat antibody (AB1128; EMD-Millipore) diluted 1:500 in blocking buffer as a primary antibody. Immune detection was performed using an HRP-conjugated rabbit anti-goat antibody (ab97105; Abcam, MA) as a secondary antibody. Immunostaining was performed using a DAB (3,3-diaminobenzidine) peroxidase (HRP) substrate kit (SK-4100; Vector Laboratories, CA), and plaques were counted using a light microscope with a 4× to 20× lens objective.

### Murine model for immunization and RSV infection.

BALB/c mice (Mus musculus; 6-week-old females from Charles River) were housed at the Department of Comparative Medicine, New York Medical College (Valhalla, NY). Mice were anesthetized with ketamine (100 mg/kg)-xylazine (10 mg/kg) administered via intraperitoneal injection before immunization or blood collection. Mice were immunized by intramuscular (i.m.) injections of 50 μl of the postfusion, prefusion, or combo VLP vaccine, the FI-RSV, and a placebo control (*n* = 10 per group). Immunizations were administered on days 1 and 14, and each VLP vaccine dose contained 4 μg of total recombinant RSV F admixed in a 1:1 volume with a squalene-based oil-in-water nanoemulsion (AddaVax; InvivoGen, CA). The placebo group received PBS admixed with AddaVax at a 1:1 volume. Serum was collected by retro-orbital bleeding before and after immunization. Mice were challenged with 1 × 10^6^ PFU of RSV strain A2 contained in 50 μl administered via the intranasal route as small drops (Pipetman with an ultraslim tip) at day 28. Groups of mice were euthanized at 4 and 7 days postchallenge for lung and blood harvest (see Fig. S2 in the supplemental material).

### Pulmonary RSV quantification by plaque assay.

Lungs from RSV-infected mice were harvested, weighed, and homogenized using an Omni tissue homogenizer (Omni International) in Opti-MEM I medium containing 25% sucrose, penicillin-streptomycin-glutamine (Life Technologies), and 2.5 μg/ml amphotericin B (Fungizone) (Chem-Impex International Inc., IL). Lung supernatants were obtained by centrifugation, and viral titers were measured by plaque assay as described above.

### Neutralization assay.

Plaque reduction neutralization tests (PRNT) were performed in duplicate using serum samples collected before viral challenge (day 28). Serial dilutions of serum were incubated with 100 PFU of RSV strain A2 virus for 1 h at 37°C, and the neutralization power was measured by a plaque assay in HEp-2 cells as described above. The 50% inhibitory concentration (IC_50_) calculation was performed by probit analysis (15).

### Luminex cytokine and chemokine analyses.

The magnetic bead-based sandwich immunoassays for cytokines using the MILLIPLEX MAP multiplex mouse cytokine panel 1 (EMD-Millipore) were performed according to the manufacturer's instructions. The lung samples (25 μl) were analyzed in duplicate wells using a Luminex MagPix (Luminex Corp., TX). The cytokine concentrations were determined by Luminex xPONENT v4.2 and EMD-Millipore MILLIPLEX Analyst v5.1 using 5-p log analysis. The gamma interferon (IFN-γ) analysis in lung fluids was confirmed using an enzyme-linked immunosorbent assay (ELISA) kit (eBioscience, CA).

### ELISA analysis of IgG subtypes.

Each well of the ELISA plates (Corning Costar, NY) was coated with RSV strain A2 containing 100 ng of F protein content determined by dot blot analysis using purified recombinant F protein (Sino Biological, PA) as the standard and incubated at 4°C overnight (see above). Serum samples were serially diluted in blocking buffer (5% milk in TBS-Tween), applied in triplicate to the ELISA plates and incubated for 2 h at room temperature. Following washes, detection was carried out using the following antibodies: IgG1 subtype (115-035-205; Jackson ImmunoResearch Laboratory, PA) and IgG2a subtype (115-035-206; Jackson ImmunoResearch Laboratory). The ELISA measurements were performed using the ECL method and a microplate reader (Synergy H1; BioTek, VT). The endpoint calculation for the ELISA was performed according to the method of Frey et al. (16).

### Histopathology.

Lungs were harvested on day 4 postinfection and fixed in 10% buffered formalin phosphate. The lung samples were processed at the Department of Pathology, New York Medical College (Valhalla, NY) and stained with hematoxylin and eosin (H&E) following a standard protocol (17). The examination and scoring of the lung histopathology were performed by blind evaluation of the H&E slides.

### Ethics statement.

The Institutional Animal Care and Use Committee (IACUC) of the New York Medical College approved the protocol for the mouse study. The study was performed in compliance with the approved IACUC protocol (no. 33-2-0513H) and the Institutional Biosafety Committee policies and under strict accordance to the Office of Laboratory Animal Welfare (OLAW) guidelines and the Public Health Service (PHS) Policy on Humane Care and Use of Laboratory Animals (NIH).

### Statistics.

Data were statistically analyzed and graphed using GraphPad Prism (GraphPad Software, CA), and errors bars represent the calculated standard errors. The statistical significance of the data was measured by one-way analysis of variance (ANOVA) with Dunnett's multiple comparisons between experimental conditions and *t* tests. The ratio of IgG2a to IgG1 was analyzed using the Taylor expansion statistical approach for calculating standard errors. Pictures and images were processed using Adobe Photoshop (Adobe Systems, CA).

## RESULTS

### Development of recombinant RSV F exhibiting postfusion and prefusion conformations.

The structural analysis of the postfusion F shows that the C terminus of F2 is in the opposite orientation and distant from the N terminus of F1 (Fig. 1B), whereas these domains are adjacent in the prefusion conformation (9, 13). We identified within this region several amino acids that are in close proximity, separated by <10 Å. Based on this analysis, we generated 9 recombinant constructs with alternative disulfide bonds between these domains to stabilize F in its prefusion conformation and 1 with the furin cleavage site mutated (see Table S1 in the supplemental material). Prefusion F VLPs were produced in mammalian cells and analyzed by dot blots with the monoclonal antibodies (mAbs) palivizumab (antigenic site II) to measure F protein expression and 5C4 to assess the presence and stability of the antigenic site Φ (see Table S1 and Fig. S1 in the supplemental material). We evaluated the postfusion state with the mAb 131-2a, which is specific for the antigenic site I (see Table S1). We found that the most stable prefusion F contained an intrachain disulfide bond inside the F1 subunit described by McLellan et al. (S155C/S290C) (18) plus an interchain disulfide bond between the F1 and F2 subunits with the cysteine substitutions A102C and I148C (Fig. 1A and B). By dot blot analysis with 5C4, we found that the prefusion F recombinant is recognized 19.2 ± 2.4-fold more than the postfusion F (Fig. 1C). To assess whether the disulfide bridge A102C/I148C enhanced the stability of F prefusion, we tested an intermediate mutant having only one cysteine change (A102C). Indeed, the mutant A102C construct demonstrated reactivity with 5C4 equivalent to that of the wild-type F postfusion construct. In addition, the dot blot analysis demonstrated that the combination of disulfide bonds, S155C/S290C with A102C/I148C, enhances the 5C4 reactivity with respect to the single disulfide bond constructs (see Fig. S1). VLPs assembled with this mutant (S155C/S290C plus A102C/I148C) were used in the vaccine studies. In addition, this F construct contains the cytoplasmic tail domain of human metapneumovirus (hMPV) F (Fig. 1A), which seems to further stabilize the structure of F incorporated in the particles as reflected by strong reactivity with 5C4 and palivizumab (Fig. 1C). This and other mutants without the hMPV F tail demonstrated strong reactivity with 5C4 but weaker reactivity with palivizumab (see Table S1).

### Generation of VLPs displaying RSV F postfusion or prefusion conformations.

**FIG 2:**
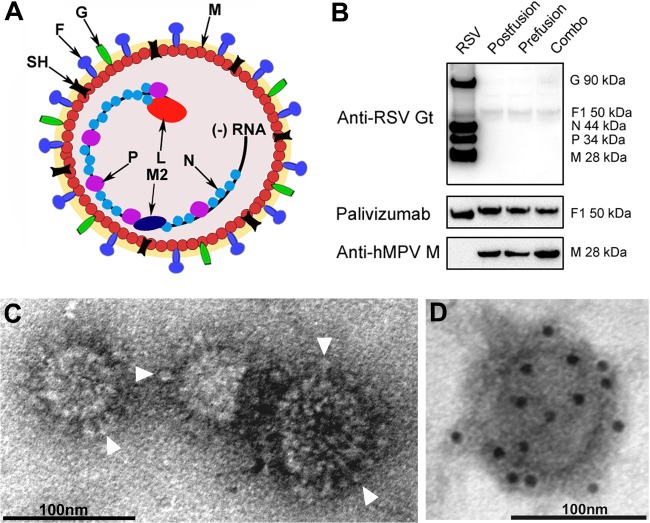
Structure and morphology of RSV VLPs using recombinant RSV F and hMPV M. (A) Drawing of RSV viral particles. F, G, SH, M, M2, P, N, and L proteins and genomic negative-sense RNA are indicated. (B) Western blot analysis of RSV VLPs using different antibodies such as goat anti-RSV (Anti-RSV Gt), palivizumab, and anti-hMPV M. The Western blotting experiment includes a purified RSV control. (C) Purified RSV VLPs were negatively stained and examined by electron microscopy. The micrograph shows spherical and irregular particles (90 to 100 nm) decorated with surface projections or “spikes” (arrowheads) resembling the morphology of RSV F. (D) Electron micrograph of immunogold-labeled RSV VLPs probed with the humanized monoclonal antibody palivizumab and developed with a goat anti-human antibody coupled to gold spheres (10 nm). The detection of gold spheres demonstrates that F is decorating the surface of the VLPs.

The RSV envelope displays three virally encoded and membrane-anchored proteins, F, G, and SH (Fig. 2A) (19). Underlying the envelope is the matrix protein (M), which during morphogenesis multimerizes and drives virion assembly and budding (20). To assemble VLPs, we utilized the RSV F either postfusion or prefusion together with the M of the hMPV as the scaffold, which we found to be more efficient than the RSV M in VLP formation (data not shown). To optimize the interaction of RSV F and hMPV M, we replaced the cytoplasmic domain of the RSV F protein with the analogous domain of the hMPV F protein, which, based on yield analysis in comparison to the unmodified F, demonstrated enhanced RSV F recruitment and incorporation onto the VLP surface (Fig. 1C and data not shown).

The analysis of purified VLPs by Western blotting showed that the RSV F hybrid copurified with the hMPV M (Fig. 2B) and that replacement of the cytoplasmic tail enhanced incorporation of the RSV F into particles compared to that for wild-type RSV F (data not shown). These results suggest that the RSV F hybrids interact with the hMPV M via its engineered cytoplasmic domain. Examination of purified VLPs by electron microscopy (EM) showed spherical structures of ∼80 nm in diameter that display F spikes protruding from the membrane envelope (Fig. 2C and D). Immunogold labeling for EM confirmed that the spikes were indeed composed of the glycoprotein F (Fig. 2D).

### Efficacy evaluation of RSV F VLP vaccines in a murine model.

**FIG 3:**
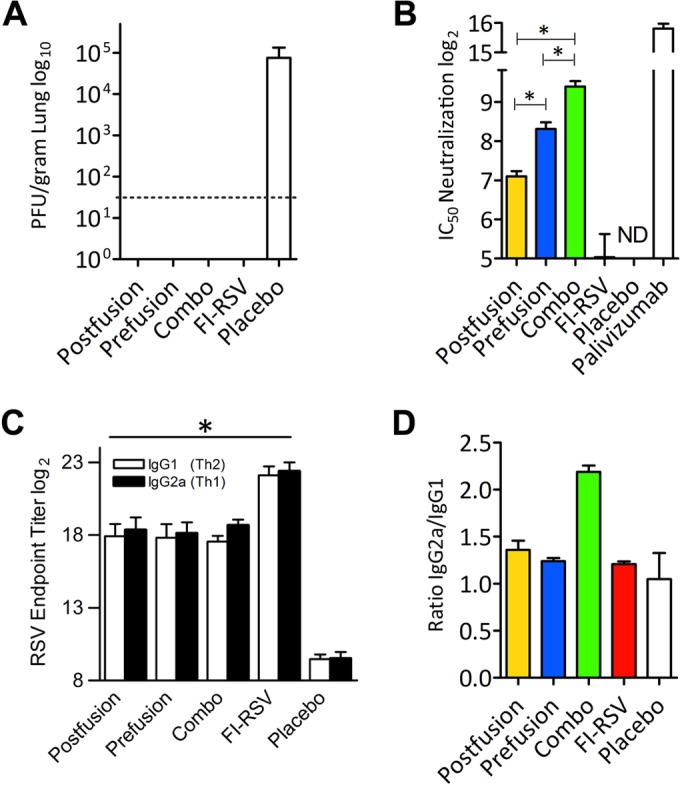
VLP vaccine protects against RSV infection. (A) Plaque assay analysis of viral titers in mouse lungs 4 days postchallenge shows undetectable viral replication in VLP-vaccinated mice, whereas the placebo control group demonstrates a very productive infection. The dotted line indicates the lower detection limit. (B) The viral microneutralization assay shows the levels of serum-neutralizing antibodies after vaccination. The combo VLP vaccination resulted in a statistically significant enhancement of neutralizing antibodies with respect to VLP postfusion and prefusion vaccination (*, *P* < 0.05). ND, not detectable. (C) Measurement of RSV-specific IgG1 and IgG2a serum antibody titers. Serum samples collected on day 28 postimmunization (*n* = 4 per group) were assayed for RSV-specific IgG isotypes by ELISA. The results are presented as the reciprocal of the log_2_ serum endpoint dilution. The antibody titers in the vaccinated mice are statistically significant with respect to the placebo control (*, *P* < 0.05). (D) Analysis of the ratio of IgG2a to IgG1 demonstrates that the combo VLP vaccine induces a superior Th1-mediated response. The results were generated using 4 mice for each condition.

To assess the protective efficacy of the VLP vaccines, BALB/c mice were immunized twice with formulations containing the postfusion F VLP vaccine, the prefusion F VLP vaccine, or a combination of the two VLP vaccines (combo VLP vaccine) and then challenged with RSV (see Fig. S2 in the supplemental material). To evaluate the safety of the VLP vaccines, we included a group of mice immunized with the FI-RSV vaccine, expected to induce vaccine-enhanced disease. The analysis of the protective efficacy on day 4 postchallenge showed that the VLP-immunized mice were completely protected from RSV replication and did not show a detectable viral load (<50 PFU/gram of lung tissue), whereas the placebo group demonstrated high levels of infective particles inside their lungs (75,000 PFU/gram of lung tissue) (Fig. 3). The assessment of the viral load on day 7 postchallenge demonstrated the absence of replicating virus in the lungs of all of the animals (data not shown). To appraise the quality and magnitude of the antibody response, we measured the serum-neutralizing activity of the immunized animals prior to viral challenge (day 28) by plaque reduction neutralization tests (PRNT) (Fig. 3B). This analysis showed that the combo VLP vaccine (postfusion plus prefusion F) elicited the highest level of neutralizing antibodies compared to either the postfusion or prefusion F single vaccine formulations. The prefusion F VLP vaccine, however, elicited higher neutralizing antibody titers than the postfusion vaccine, results that agree with previous reports (21, 22). On the other hand, the FI-RSV vaccine failed to induce an appreciable level of neutralizing antibodies. The palivizumab control demonstrated an IC_50_ neutralizing activity of 2 μg/ml, a value that is similar to that for previous determinations (23).

### VLP vaccine stimulates a balanced IgG response.

We assessed the magnitude of the IgG2a and IgG1 serum responses, which are correlates of Th1 and Th2 development, respectively (Fig. 3C and D). Mice that received the VLP vaccines demonstrated induction of robust serum IgG responses compared to those receiving the preimmune samples. On the other hand, serum from FI-RSV-immunized mice showed the highest level of IgG induction, suggesting that antibodies to multiple viral proteins were produced and detected in the whole-virus ELISA. The analysis of the IgG subtype demonstrated that the combo VLP vaccine elicited a balanced Th1-mediated versus Th2-mediated response, associated with a greater ratio of IgG2a to IgG1 (Fig. 3D). As expected, the placebo control demonstrated background levels of total and specific IgGs against RSV.

### Analysis of the cytokine profile in VLP-vaccinated and control mice after RSV infection.

**FIG 4:**
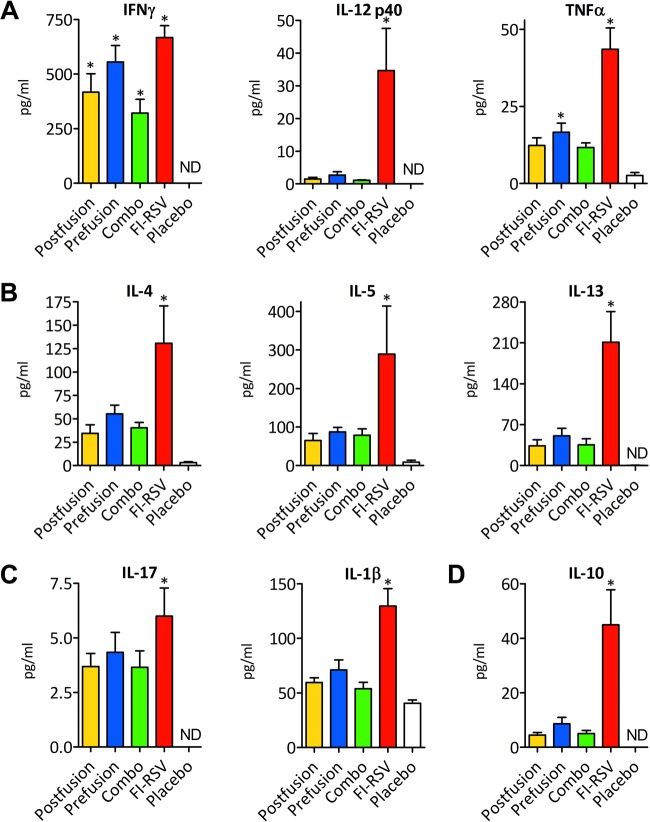
Cytokine responses in VLP-vaccinated mouse lungs after RSV infection. (A) The cytokines for the Th1-mediated response are IFN-γ, IL-12p40, and TNF-α. (B) The Th2-mediated response cytokine measurement includes IL-4, IL-5, and IL-13. (C) The IL-17 and IL-1β analysis indicates the Th17-mediated response. (D) The IL-10 cytokine analysis demonstrates immunoregulatory process development. The results were generated using a group of 4 mice for each condition at 4 days postinfection. An asterisk indicates a statistically significant difference between the experimental condition and the placebo control; differences between the FI-RSV and VLP vaccines were statistically significant (*P* < 0.05) in all groups, with the exception of the prefusion group in the IFN-γ assay. ND, not detectable.

We applied Luminex technology to study the cytokine and chemokine levels in lung homogenates of VLP-vaccinated and control mice 4 days after challenge. We evaluated the cytokine markers that correlate with the Th1, Th2, and Th17 types of immune responses as well as interleukin-10 (IL-10) (Fig. 4A to D). VLP-immunized mice showed a robust IFN-γ response in comparison to that of the placebo control (Fig. 4A). On the other hand, immunization with the FI-RSV vaccine stimulated a strong cytokine response that qualitatively and quantitatively differed from the one elicited in mice immunized with the VLP vaccine or placebo. We found that FI-RSV immunization induced high levels of the cytokines IFN-γ, tumor necrosis factor alpha (TNF-α), IL-4, IL-10, IL-17, and IL-1β, all of which have been associated with the exacerbation of RSV disease (24–29) (Fig. 4A to D). In contrast, multiplex analysis of the placebo control demonstrated very low expression levels of these cytokines, indicating that RSV replication did not trigger or perhaps curtailed production of these immune-signaling molecules (Fig. 4A to D). This is in agreement with the results of Lambert and coworkers (30), who also found very low levels of IFN-γ, IL-5, IL-13, and IL-17 in the lungs of the placebo group on day 4 postchallenge. However, higher doses of the challenging virus (e.g., 1 × 10^7^ PFU) increase the levels of the secreted cytokines as described by Rutigliano and Graham (29).

**FIG 5:**
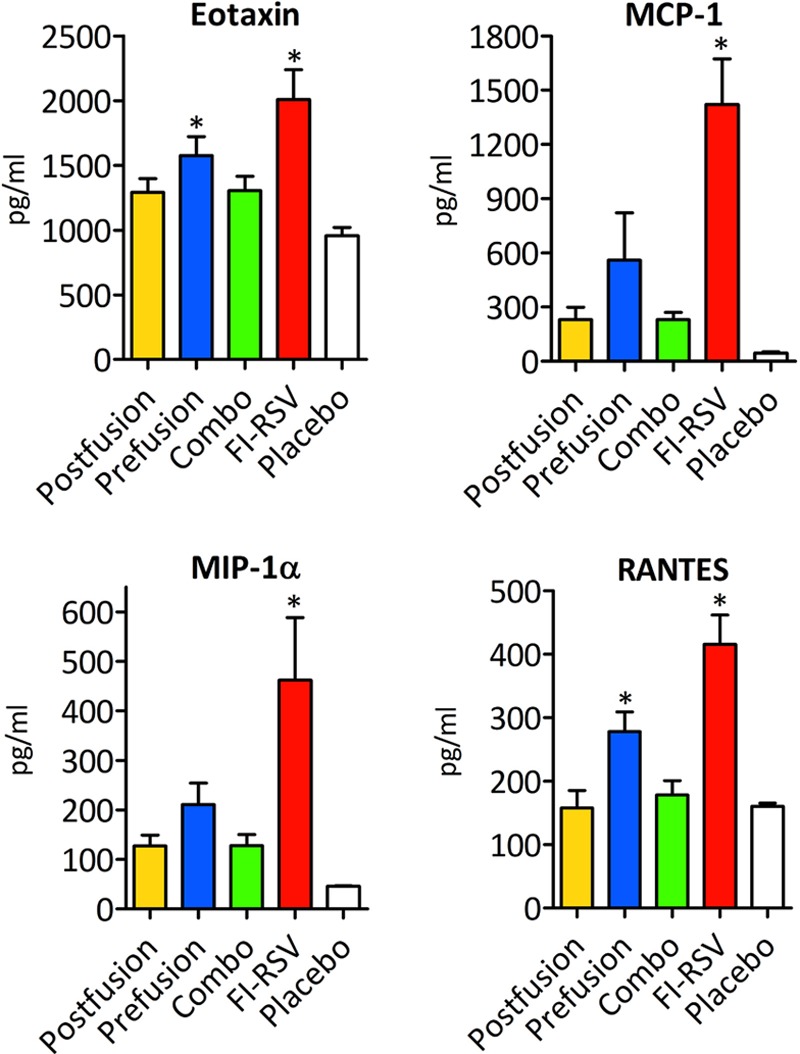
Chemokine responses in VLP-vaccinated mouse lungs after RSV infection. Analyses include eotaxin, MCP-1, MIP-1α, and RANTES. The results were generated using a group of 4 mice for each condition at 4 days postinfection. An asterisk indicates a statistically significant difference between an experimental condition and the placebo control (*P* < 0.05). Differences between the FI-RSV and the VLP vaccines are also statistically significant (*P* < 0.05) in all groups.

Chemokines recruit inflammatory cells to the infected tissue and are particularly elevated in bronchiolitis (31, 32). Thus, we analyzed eotaxin, monocyte chemoattractant protein 1 (MCP-1), macrophage inflammatory protein 1 alpha (MIP-1α), and RANTES, all of which are involved in lung immune cell infiltration during bronchiolitis (Fig. 5). While FI-RSV vaccine strongly augmented each chemokine, the VLP-immunized animals had a less significant induction of these inflammatory mediators, the levels of which were closer to those seen in the placebo controls.

### Evaluation of VLP vaccine safety by lung histopathology examination.

**FIG 6:**
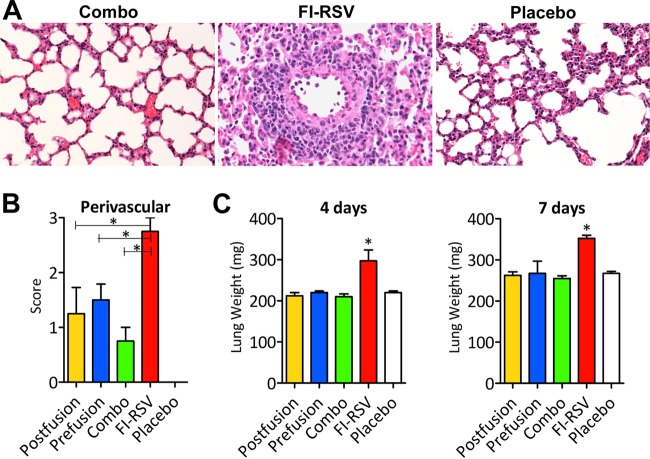
RSV VLP vaccines do not induce “vaccine-enhanced disease” in murine lung. (A) Hematoxylin and eosin staining of mouse lung 4 days after viral challenge. Microscopic examination of the lungs after the combo VLP vaccination shows the absence of or minor pulmonary pathology. On the other hand, the FI-RSV vaccination induces a very high perivascular immune infiltration. A placebo control is included as a reference. (B) Perivascular infiltration was scored by blind evaluation of sections of mouse lung stained with hematoxylin and eosin 4 days after the viral challenge. The scores range from 0 for normal to a maximum of 3 for massive infiltration. An asterisk indicates a statistically significant difference between VLP vaccination and FI-RSV (*P* < 0.05). (C) Mouse lungs were harvested 4 and 7 days after RSV challenge and weighed. An asterisk indicates a statistically significant difference with respect to the VLP vaccines and placebo control (*P* < 0.05).

VLP vaccine safety and tolerability were further evaluated by histological examination of the lung tissues of VLP-vaccinated mice at 4 days postinfection (Fig. 6A and B). The placebo control mice that received a primary infection with 10^6^ PFU of RSV experienced some interstitial cellular infiltrate but limited or no sign of perivascular infiltration on day 4 postchallenge, indicating that virus replication was tolerated without provoking serious lesions. Indeed, a previous report (30) has shown minimal eosinophilic infiltration in primary infected mice under the same experimental conditions.

FI-RSV-vaccinated mice displayed massive perivascular, peribronchial, and interstitial infiltrations of inflammatory cells. In contrast, the VLP-immunized mice showed limited immune cell infiltrations. Blinded scoring of the perivascular infiltration demonstrated that the combo VLP vaccine had the lowest level of histological changes (Fig. 6B). The perivascular infiltrations in the postfusion and prefusion vaccine groups were more pronounced than those of the placebo controls; however, the overall lung architectures were not significantly different among these groups. Severe RSV disease and FI-RSV vaccine-enhanced disease are characterized by cellular infiltration and lung hyperinflation (33, 34). We found that the lungs from mice immunized with FI-RSV were significantly larger and were >30% heavier than the lungs from the placebo controls, while the lungs from the VLP-vaccinated mice did not differ from those of the placebo group (Fig. 6C).

## DISCUSSION

Here we present data on the immunogenicity, efficacy, and safety of a novel VLP-based RSV vaccine constructed with different conformations of the RSV F glycoprotein. Previously tested subunit vaccines were formulated primarily with RSV F in its postfusion conformation (35, 36). Recent studies (21, 22, 37), however, have demonstrated that the RSV F in the prefusion conformation has the ability to elicit higher levels of neutralizing antibodies than the postfusion conformation. Indeed, different groups have shown that vaccines containing RSV F in the prefusion conformation were superior for protecting mice and cotton rats from RSV infection (21, 22, 37). Considering these data, we designed, produced, and tested a novel recombinant stabilized prefusion F that is highly expressed in mammalian cells, is incorporated into VLPs, and is recognized strongly by the mAb 5C4, which binds the prefusion-only antigenic site Φ.

Although VLPs are strong immunogens, we included an adjuvant in the VLP vaccine in order to elicit the greatest immunogenicity. Protection against RSV infection may require a greater immunity than that stimulated by natural infection, which does not prevent reinfection. We selected the squalene-based oil emulsion because it is a potent inducer of both Th1- and Th2-mediated immunity and is well tolerated and safe (38, 39).

Assessment of the protective efficacy afforded by VLP vaccination after RSV challenge showed that each one of the three VLP vaccine formulations protected the lungs from viral infection. Furthermore, evaluation of the serum neutralization potency showed that the prefusion F VLP vaccine induced antibodies with a higher neutralization power than did immunization with the postfusion F VLP vaccine, in agreement with the results of previous studies (21, 22), although not to the same extent. However, the combo VLP vaccine showed the best neutralization activity, reaching a neutralizing power that was >4-fold greater than that seen with the postfusion VLP vaccine and >2-fold greater than that seen with the prefusion VLP vaccine. Notably, all VLP vaccines contained the same total F protein content (4 μg total), suggesting that the combination of the two conformations of F protein may be synergistic in eliciting a protective immune response. Recent studies performed with the Newcastle disease virus VLP vaccines showed similar results when the postfusion and prefusion forms were compared; however, a combo formulation was not tested (21, 40). It seems reasonable to speculate that the combo VLP vaccine displays a larger repertoire of neutralizing epitopes than either of its components. This outcome is significant for RSV vaccine development and requires further investigation to define the underlying mechanism.

Consistent with the antibody neutralization assay data, the IgG isotyping analysis showed that the combo VLP vaccine induced a balanced Th1-mediated immune response. On the other hand, the FI-RSV vaccine elicited high levels of IgG that did not correlate with the induction of neutralizing antibodies. This outcome clearly illustrates the dichotomy between high antibody titers and neutralizing capacity, which has been the hallmark of the FI-RSV vaccine-enhanced disease.

The cytokine analyses showed that all VLP-vaccinated mice produced statistically significant levels of IFN-γ compared to those of the placebo group, which is a correlate of induction of a Th1 type of immune response. On the other hand, the FI-RSV vaccine induced high levels of IFN-γ and TNF-α, as well as high production of Th2-polarizing cytokines (IL-4, IL-5, and IL-13), Th17-polarizing cytokines (IL-17 and IL-1β) and IL-10, and chemokines (eotaxin, MCP-1, MIP-1α, and RANTES), all of which have been associated with enhanced RSV pathogenesis (24–29). In contrast, VLP-vaccinated mice produced much smaller amounts of these inflammatory cytokines and chemokines. Furthermore, histopathology studies showed that VLP vaccination did not induce the detrimental immune cell infiltration inside the lung and that the combo VLP formulation was, in this regard, the best-tolerated vaccine.

In summary, we describe the production of RSV VLPs composed of RSV F glycoprotein that display different epitopes suitable for the elicitation of neutralizing antibodies. Considering the diversity, neutralizing strength, and distribution of epitopes, both shared and unique, between the two conformations of F, it seemed important to compare the immunogenicity and efficacy of single VLP vaccines (postfusion or prefusion F) with those of a combo VLP formulation. This study showed that the combo VLP vaccine, composed of the multiple epitopes revealed in the postfusion and prefusion F, afforded complete protection against RSV infection and elicited production of the highest level of serum-neutralizing antibodies that correlate with the development of a strong Th1 immune response. Furthermore, immunization with this vaccine proved to be safe, a condition that must be satisfied by any RSV vaccine candidate. We anticipate that the combo VLP vaccine may elicit a broader spectrum of neutralizing antibodies and thus afford better protection against RSV infection and is a viable safe and efficacious candidate for clinical evaluation.

## Supplementary Material

Supplemental material
