# Supplementary material for: Novel Respiratory Syncytial Virus-Like Particle Vaccine Composed of the Postfusion and Prefusion Conformations of the F Glycoprotein
Source: Clin Vaccine Immunol. 2016 Jun 6;23(6):451–9. doi: 10.1128/CVI.00720-15 (PMC4895010; doi:10.1128/CVI.00720-15)
Supplement: Supplemental material [file supp_23_6_451__index.html]

Supplemental material 

# Novel Respiratory Syncytial Virus-Like Particle Vaccine Composed of the Postfusion and Prefusion Conformations of the F Glycoprotein

## Supplemental material

- Supplemental file 1 -

  Table S1. List of prefusion F constructs generated by mutagenesis. Fig. S1. 5C4 immunoreactivity of RSV F prefusion constructs. Fig. S2. Diagram of the vaccination schedule used in BALB/c mice.

  PDF, 161K
