# Supplementary material for: Novel Respiratory Syncytial Virus-Like Particle Vaccine Composed of the Postfusion and Prefusion Conformations of the F Glycoprotein
Source: Clin Vaccine Immunol. 2016 Jun 6;23(6):451–9. doi: 10.1128/CVI.00720-15 (PMC4895010; doi:10.1128/CVI.00720-15)
Supplement: Supplemental material [file CVI.00720-15_zcd999095353so1.pdf]

SUPPLEMENTAL MATERIAL

F Constructs	5C4	131-2A	Palivizum.
Wild Type	1.0	1.0	1.0
S155C-S290C (McLellan et al)	8.0	0.1	0.1
A102C-I148C	3.2	0.5	1.0
N105C-G145C	ND	ND	ND
N105C-I148C	3.7	0.4	0.6
F32C-L467C	0.1	ND	ND
E30C-L467C	ND	ND	ND
F32C-Y468C	ND	ND	ND
F32C-V469C	0.1	ND	ND
A102C-I148C, S155C-S290C	13.9	0.1	0.1
Furin Site 1* and Furin Site 2[§]	2.3	0.7	0.7
Furin Site 1* and Furin Site 2[§], A102C-I148C	0.7	0.2	0.1

* Mutations Furin Site 1: R133K R135H R136Q

[§] Mutations Furin Site 2: R106K R108H R109Q

Supplemental Table 1) List of prefusion F constructs generated by mutagenesis.

RSV F constructs were tested by immunoblot analysis. The presence and quantity of the prefusion epitope ϕ was determined using the 5C4 antibody. The presence of the postfusion antigenic site I was assessed using the 131-2a antibody, and the level of protein expression was determined using the palivizumab antibody. Quantification is normalized with respect to the immunoreactivity of the wild type RSV F construct

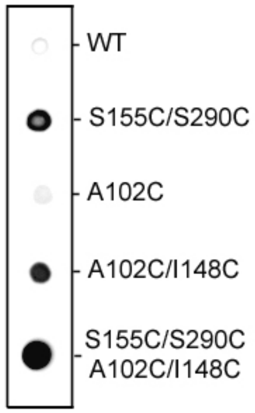

Supplemental Figure 1) 5C4 immunoreactivity of RSV F prefusion constructs

Dot blot analysis showing the immune reactivity of recombinant RSV F proteins with combination of cysteine mutations. To assess whether the disulfide bridge A129C/I148C enhanced the stability of F provided by S155C/S290C, we tested an intermediate mutant having S155C/S290C and only one cysteine change, A129C. This construct demonstrated reactivity with 5C4 equivalent to S155C/S290C, and much lower than the F that contained both disulfide bridges (S155C/S290C) and A102C/I148C.

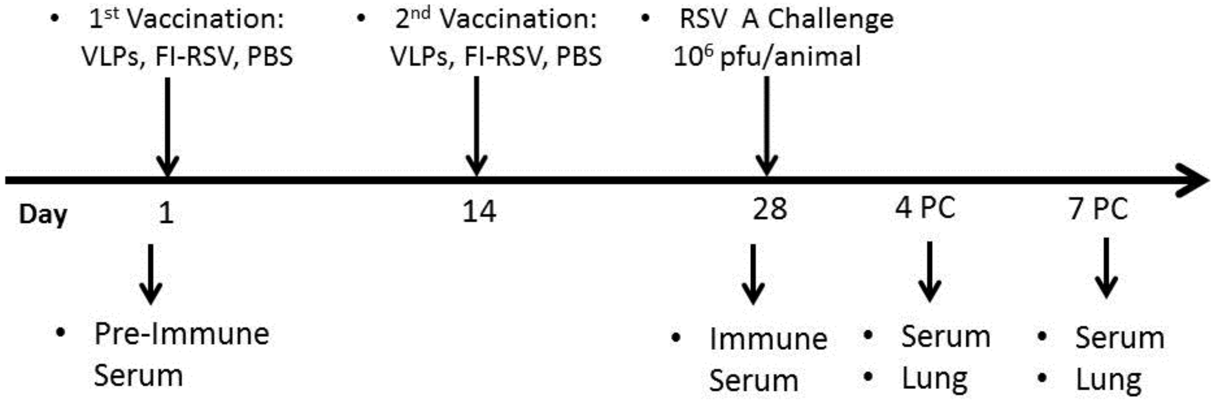

Supplemental Figure 2) Diagram of the vaccination schedule used in BALB/C mice

Sero-negative mice were immunized by intramuscular injection at day 1 and 14. Mice were challenged with $1x10^6$ pfu of RSV A Long strain administered via the intranasal route at day 28; animals were sacrificed at days 4 and 7 post RSV-challenge (PC).
